# Mammography: an update of the EUSOBI recommendations on information for women

**DOI:** 10.1007/s13244-016-0531-4

**Published:** 2016-11-16

**Authors:** Francesco Sardanelli, Eva M. Fallenberg, Paola Clauser, Rubina M. Trimboli, Julia Camps-Herrero, Thomas H. Helbich, Gabor Forrai, for the European Society of Breast Imaging (EUSOBI), with language review by Europa Donna–The European Breast Cancer Coalition

**Affiliations:** 1 Department of Biomedical Sciences for Health, University of Milan, Milan, Italy; 2 Department of Radiology, Research Hospital (IRCCS) Policlinico San Donato, San Donato Milanese, Milan, Italy; 3 Department of Radiology, Charité-Universitätsmedizin Berlin, Campus Virchow-Klinikum, Berlin, Germany; 4 Department of Biomedical Imaging and Image-guided Therapy, Division of Molecular and Gender Imaging, Medical University of Vienna, Vienna, Austria; 5 Integrative Biomedical Research PhD Program, Università degli Studi di Milano, Milan, Italy; 6 Department of Radiology, Hospital de la Ribera, Alzira, Valencia Spain; 7 Department of Radiology, Duna Medical Center, Budapest, Hungary

**Keywords:** Breast cancer, Mammography, Screening, Digital breast tomosynthesis (DBT), Contrast-enhanced spectral mammography (CESM)

## Abstract

**Abstract:**

This article summarises the information to be offered to women about mammography. After a delineation of the aim of early diagnosis of breast cancer, the difference between screening mammography and diagnostic mammography is explained. The need to bring images and reports from the previous mammogram (and from other recent breast imaging examinations) is highlighted. Mammography technique and procedure are described with particular attention to discomfort and pain experienced by a small number of women who undergo the test. Information is given on the recall during a screening programme and on the request for further work-up after a diagnostic mammography. The logic of the mammography report and of classification systems such as R1-R5 and BI-RADS is illustrated, and brief but clear information is given about the diagnostic performance of the test, with particular reference to interval cancers, i.e., those cancers that are missed at screening mammography. Moreover, the breast cancer risk due to radiation exposure from mammography is compared to the reduction in mortality obtained with the test, and the concept of overdiagnosis is presented with a reliable estimation of its extent. Information about new mammographic technologies (tomosynthesis and contrast-enhanced spectral mammography) is also given. Finally, frequently asked questions are answered.

***Key Points*:**

• *Direct digital mammography should be preferred to film-screen or phosphor plates*.

• *Screening (in asymptomatic women) should be distinguished from diagnosis (in symptomatic women)*.

• *A breast symptom has to be considered even after a negative mammogram*.

• *Digital breast tomosynthesis increases cancer detection and decreases the recall rate*.

• *Contrast-enhanced spectral mammography can help in cancer detection and lesion characterisation*.

## Introduction

Malignant tumours (cancers) and benign diseases are very common in the breast. Aside from clinical history (disorders in the family, previous breast diseases/surgery, hormone therapy, personal well-being and complaints), inspection (external viewing) and palpation, which compose the so-called clinical breast examination, imaging procedures and especially mammography are of crucial importance in the detection and diagnosis of breast cancer and also other breast diseases. Mammography is a specialised radiography of the breast using x-rays for generating images of the breast. Its purposes are first early detection of breast cancer before symptoms (screening mammography) and second diagnosis in patients with symptoms such as a palpable lump (*diagnostic mammography*, also named *clinical mammography*).

This article—specifically aimed at summarising the most important information to be offered to women about mammography—updates a previous article published in 2012 [1] by the European Society of Breast Imaging (EUSOBI), taking in consideration the most recent evidence in favour of mammography and of two mammographic technique tools now available for clinical practice: digital breast tomosynthesis (or simply *tomosynthesis*) and *contrast-enhanced spectral mammography* (CESM). Here we also took into account the recent position paper on screening for breast cancer by EUSOBI and 30 national breast radiology bodies [2], which should be considered complementary to the current article.

## Screening and diagnostic mammography

Mammography is the most important imaging procedure for breast cancer detection and diagnosis. The general aim is to enable early treatment of breast cancer, to improve survival rates and to reduce the need for aggressive treatment such as mastectomy [3, 4], also in the current era of modern therapies [5, 6]. It can be performed in a screening setting or a diagnostic setting. In both settings, whenever possible, *preference should be given to full-field digital mammography (not phosphor-plate computer radiography) instead of film-screen mammography*, taking in consideration a number of relevant advantages for the women who get a mammogram and for the general population, including lower x-ray dose, higher image quality, possibility of post-processing, digital archiving, image transmission and no chemical pollution [2, 7].

### Screening mammography

Screening is performed periodically in order to find small cancers before they are detected through self-palpation or clinical breast examination. Mammography is performed every 1, 2 or 3 years from the age of 40–50 years until around 70–75, depending on national/regional screening programmes. European guidelines suggest the 2-year interval for the general female population from 50 to 70 years of age [8]. Relevant differences in screening programmes across European countries, including ways of reporting, are due to differences in culture, technical circumstances, biopsy options, financial restrictions and breast cancer prevalence. Women with a high frequency of breast cancer in their family should start even earlier with periodic imaging, possibly with protocols including contrast-enhanced magnetic resonance imaging (MRI) [9, 10], after consulting specialised centres, since mammograms in those conditions may have a very limited diagnostic power.

Screening mammography is a standardised procedure composed of four views (also named projections), two for each breast: the cranio-caudal projection and the medio-lateral oblique projection. In some countries, clinical breast examination is a part of the procedure, even though its added value in the screening setting, when mammography is performed, is negligible [4]. Screening mammography can be performed by a radiographer alone; the resulting images are usually read by two radiologists, independently, in separate sessions. If the examination is judged to not reveal any abnormality suspicious for malignancy, the woman receives a letter communicating this result. If something suspicious is found, the woman is recalled for a tailored further assessment that can be variably composed of additional mammographic views, tomosynthesis, ultrasound, MRI, CESM or needle biopsy. When this assessment is concluded, a formal written report will be prepared by the radiologist and given to the woman during a dedicated interview for complete information.

### Diagnostic mammography

This is performed in patients presenting with clinical symptoms such as a palpable lump, nipple discharge, skin thickening and/or nipple retraction in order to diagnose or exclude breast cancer. Diagnostic mammography is usually performed by a radiographer and images are immediately available for the radiologist to assess. Before or after the bilateral acquisition of the two standard projections already mentioned for screening mammography, a full clinical breast examination is performed by the radiologist. This is particularly important when results of a full clinical breast examination recently performed by another doctor are not available. According to the radiologist’s preference, palpable lumps, scars from previous surgeries or other abnormalities can be highlighted by positioning a marker on the skin. If necessary, additional views are acquired after the standard procedure, and further assessment can be performed as above described for women with suspicious findings at screening mammography. A formal written report is always prepared by the radiologist with conclusions, including recommended further steps.

Note A. If you notice relevant symptoms in your breast, ask immediately for an appointment with your primary care physician in order to decide if you need a diagnostic mammography. Alternatively, you may also directly ask your breast radiologist for a prompt evaluation. This advice is also valid even if you recently had a screening mammography that did not reveal suspicious findings. However, if you have symptoms and you are getting a screening mammography, inform the radiographer about them! The radiologists reading your images will be informed about this and will decide whether you should be recalled based on these symptoms. In any case, if your symptoms do not disappear, you should consult your radiologist even if your mammography has been judged negative.

## Scheduling/precautions

The best time for a less painful mammography to be carried out is from day 7 to day 12 after the beginning of the woman’s last menstruation. No particular scheduling is required after menopause, implying that for the majority of mammograms performed in the context of population-based screening programmes scheduling has no limitations. If the woman is pregnant, ultrasound is preferred as a first option.

Note B. You should bring images and reports from the previous mammograms (and from other recent breast imaging examinations) and give these to the radiographer or the radiologist before the procedure. This can be crucial for image interpretation due to the fact that some cancers are diagnosed only on the basis of changes that have occurred after a previous examination.

## Technique/procedure

Mammography is performed using a dedicated x-ray unit. A particular radiographic technique is used requiring the compression of the breast for 5–10 s in order to deliver a low radiation dose and to obtain high-quality images. As already mentioned, it is standard practice to take two views per breast and additional views in special cases. The procedure is performed with the woman’s upper body undressed. All foreign objects (such as bras, necklaces, piercings, etc.) must be removed before the procedure. The woman will stand in an upright position in front of the machine. For each projection of each breast, the radiographer will place the breast on the plate and will carefully apply a progressive compression for 5–10 s. During breast squeezing, women may feel some pain or discomfort [11]. *It is important not to move during this short time*. Immediately after acquiring the mammogram, the breast will be released from compression. The entire bilateral standard procedure, including preparation, takes approximately 5–10 min.

Note C. To reduce pain or discomfort due to breast compression and to get the best mammograms, you should relax during the procedure; in particular, the pectoral muscles should be relaxed. Follow the radiographer’s instructions exactly and bear in mind that heavier compression means a lower x-ray dose, higher image quality and easier diagnosis. If you previously experienced a painful mammography in the premenstrual phase, try to arrange the next one from day 7 to day 12 of your cycle.

## After the procedure

When the procedure is over, the woman returns to the waiting room. In the case of screening mammography, she is usually only informed whether or not the acquired images are technically adequate. If no views need to be repeated, she may leave. She will receive a letter communicating that the mammogram is negative or she will be informed, usually through a phone call, that further assessment is needed (*recall*). The first event is far more probable (over 90–95 % of cases). In some countries, only positive screening examinations (recalls) are communicated. In the case of diagnostic mammography, after checking the technical adequacy, the radiologist immediately informs the patient either that the examination is completely negative or that further assessment is needed, as already mentioned.

Note D. If you are recalled after a screening mammogram or you are asked to have an ultrasound after a diagnostic mammography, this does not mean that you have a cancer. The most probable result of this second examination, especially in the screening setting, is a higher level of certainty in stating that you do not have cancer! Less than 10 % of women recalled at screening are finally diagnosed with cancer. However, if a cancer were present, you would rightly like it to be diagnosed as early as possible.

## Mammography report and classification systems

Diagnostic mammography and also diagnostic assessment of recalled women after mammography screening should be formally carried out by a certified breast radiologist. A detailed report should include a description of the clinical context, if relevant, as well as image findings, including breast density and structure according to different classification systems, interpretation of the described findings and a final conclusion with recommendations. In many European countries, standardised classification systems for the conclusions of mammography reports are used. A European system uses the five-level scale from R1 to R5, where R stands for radiography. R1 means no abnormalities, R2 benign findings, R3 equivocal findings, R4 suspected cancer and R5 strongly suspected cancer. A system developed in the USA, the Breast Imaging Reporting and Data System (BI-RADS) [12], also used in many European countries, includes a similar scale, from BI-RADS 1 to BI-RADS 5. The main difference is for BI-RADS 3, which implies a very low probability of cancer (less than 2 %), allowing the possibility of waiting for a short interval (usually 3–6 months) before a repeat mammogram. Conversely, the R3 category indicates a probability of cancer that is higher than that of BI-RADS 3. Moreover, the BI-RADS score system also includes BIRADS 0 (examination insufficient for a diagnostic conclusion; further work-up needed) and BI-RADS 6 (evaluation of an already diagnosed cancer).

Note E. In practice, if you have an R4–R5 or a BI-RADS 4–5 finding, needle biopsy is recommended. In case of R3 or BI-RADS 3, meet your radiologist and ask for a detailed explanation of this result, of the risks and probabilities associated with different options.

## Diagnostic performance of mammography

No diagnostic test is perfect. This rule also applies to mammography. When thinking about screening, women should be aware that about 28 % of cancers can be missed [13, 14], especially in pre-menopausal women and in those with dense breasts. This means that if we consider 1000 women getting a screening mammogram, if 8–10 cancers are present, 2 or 3 can be missed, mostly because they are difficult to distinguish from normal breast tissue. Still, mammography is the best proven method for screening average-risk women.

Note F. Do not underestimate the importance of breast symptoms (especially a new palpable lump, skin/nipple retraction or nipple discharge), regardless of the timing of your last negative mammogram. Go to your radiologist and ask for a visit. Tell her/him your symptoms and she/he will decide the best course of action for you. Conversely, not all suspicious findings visualised on a mammogram are cancers: depending on the level of suspicion, cancer is confirmed in a highly variable proportion of cases. When the suspicion is confirmed after further assessment, image-guided needle biopsy is mandatory before planning any treatment.

Note G. A suspicious mammographic finding is not a confirmed cancer. However, do not postpone further assessment and, if necessary, needle biopsy.

## Radiation exposure from mammography

The radiation exposure for a mammogram is low. A study [15] reported that undergoing repeated mammograms over a time period of 34 years (annual from age 40 to 55 years and biennial from 56 to 74 years) entails a risk of radiation-induced breast cancer equal to 1 in every 1000 women screened. The risk of breast cancer in the female population of western countries is equal to at least one in every ten women. The first risk is 100 times smaller than the second, while the reduction in breast cancer mortality thanks to early detection with screening mammography is about 40 % [4]. Another study [16], applying a mortality reduction rate of 43 % as an effect of screening mammography, also considering the “minimal” risk of radiation-induced cancers, found that biennial screening mammography performed in 100,000 women age 50–69 saves 350 lives. However, for the 40–49 age range, the problem of radiation effects depends on the estimated magnitude of radiation-induced BCs in this younger age interval and must be more carefully considered. *Importantly, even in the rare case of radiation-induced breast cancer, in a screening setting most of these will be detected early and treated*. In symptomatic women, when a mammogram is necessary, the advantages always outweight the disadvanges, independently from the patient age.

## Overdiagnosis

Not all the breast cancers diagnosed with screening are aggressive and fatal cancers. In the absence of screening mammography, some breast cancers—estimated to be about 6.5 %, with a range from 1 % to 10 % [4]—would have remained totally free of symptoms because of the very slow growth of these types of lesions, which do not tend to advance outside the breast [17]. However, these cancers cannot be distinguished from those that, if left undiagnosed and untreated, would be fatal. Thus, if we want to reduce breast cancer mortality, we must accept a rate of overdiagnosed cancers with the consequence of a rate of unnecessary treatment, mainly including surgery and radiation therapy. An effective representation of the balance between early diagnosis and overdiagnosis has been provided by the Euroscreen working group [18]: for every 1,000 women screened from 50 to 69 years of age, 7–9 breast cancer deaths are avoided, 4 breast cancers are overdiagnosed, 170 women have at least one recall followed by noninvasive assessment with a negative result, and 30 women have at least one recall followed by invasive procedures with a negative result. *In practice, the probability of an individual woman’s life being saved is double that of being overdiagnosed*.

## New mammographic techniques: tomosynthesis and contrast-enhanced spectral mammography

Two further developments of digital mammography were recently introduced into clinical practice: tomosynthesis and CESM. Both techniques are intended to overcome some limitations of mammography by reducing summation effects (tomosynthesis) or by increasing contrast differences (CESM), especially, but not only, in women with dense breast tissue. In these women, tumours can be masked because of overlying breast tissue and lack of contrast to the adjacent normal breast tissue is common. So far, these techniques have mainly been proposed as an adjunct to mammography in women with inconclusive findings in their initial mammograms, with interesting results. Tomosynthesis has also been positively evaluated as a screening tool.

### Tomosynthesis

This is obtained with the same mammographic unit that acquires either the usual digital mammograms or tomosynthesis studies. The same cranio-caudal and medio-lateral oblique views are acquired for both examinations and the patient preparation and positioning are alike. The most important difference is the use of a moving x-ray source in tomosynthesis. During a tomosynthesis examination, the x-ray source moves following an arc over the breast and acquires several projections. At the end, numerous images per view are obtained, each of them showing a slice of the breast [19–21]. Tomosynthesis can be acquired as an additional imaging to the usual mammograms or it can be acquired alone. The latter protocol is possible because images very similar to the usual mammograms can be reconstructed from the tomosynthesis data set: these so-called *synthetic mammograms* can avoid the need for acquiring the original usual mammograms [20, 22]. According to the device used, radiation exposure is equal to slightly higher, as compared to mammography, but it is still within the limits recommended by international radiation safety guidelines [23]. Results of different studies comparing mammography alone with mammography with tomosynthesis demonstrated that tomosynthesis is able to significantly increase cancer detection up to 30–40 % [21].

Tomosynthesis is already used as a screening modality in the USA. In Europe, only a few centres perform tomosynthesis in organised screening programmes, mostly in the context of research programmes approved by Ethical Committees. The results of these studies are promising. Three prospective studies showed that DBT used as an adjunct [24–26] or alternative [27] to the usual digital mammograms allows for a superior diagnostic performance when compared to mammography alone. Overall, tomosynthesis provides an increase in detection rate from 0.5 to 2.7 per 1000 screened women as well as a reduction in recall rate from 0.8 to 3.6 per 100 screened women [28]. All these aspects will probably confer to tomosynthesis the status of future *routine mammography* also in the screening setting.

However, before introducing tomosynthesis in breast cancer screening outside ethically approved trials, there should be evidence for a statistically significant and clinically relevant reduction in the interval cancer rate. This cautiousness is due to the need to avoid an increase in overdiagnosis and costs. First results for a reduction from 0.7 to 0.5 interval cancers per 100 screened women were recently reported from a large study in the US [29], but further evidence is needed.

Note H. During a breast examination performed outside the screening setting, it is the choice of the radiologist whether to perform only mammography, to associate tomosynthesis and/or ultrasound, or also to perform tomosynthesis without standard mammography and to obtain reconstructed synthetic mammograms. This decision is based on various issues: the characteristics of the breast, the availability of previous examinations, the availability of technology and also the radiologist’s preference in relation to the specific case.

Note I. If you are invited to attend a screening programme where tomosynthesis is proposed in the context of a study or as routine practice, consider that the potential advantages of tomosynthesis in terms of increased cancer detection and reduced recall rate should overcome the modest increase in radiation dose.

### Contrast-enhanced spectral mammography

As with contrast-enhanced MRI, the basis of contrast-enhanced mammography is the fact that, during the development and growth of a tumour, it develops its own new blood vessels, which can be a bit *leaky*, allowing an intravenously injected contrast agent to enrich the tumour. This enhances the contrast of the tumour compared to the surrounding tissue. To be able to show this tumour contrast uptake in a mammographic image, you have to acquire two exposures of the breast within the time of one compression, each of them with a different x-ray energy composition, a technical possibility available for some new mammographic units. This results in a low-energy image, identical with a normal mammogram, and a high-energy image containing information about contrast agent distribution in the breast; the use of different energies is the reason for the denomination *spectral* mammography. Depending on the breast composition and thickness, this causes an extra radiation dose of approximately 20 %, but both images together still imply an x-ray dose below the recommended dose for mammography [30–33].

Before the acquisition of the two images started, an iodinated contrast agent has to be intravenously injected. This is usually done while the patient is seated near the mammographic unit. Two minutes after the start of the injection, the patient is guided to the mammography system and positioned similarly as with a normal mammography examination. Within roughly 5 min, the usual cranio-caudal and medio-lateral oblique views of both breasts are taken bilaterally, each of them composed by a low-energy and a high-energy image. The combination of the two images by a dedicated software allows for obtaining a new image where the presence of contrast uptake is easily recognised.

The diagnostic performance of CESM has been recently summarised by a systematic review and meta-analysis [34], i.e. a combination of the results of previously published CESM studies. The authors identified eight studies (4 prospective and 4 retrospective) for a total of 920 patients with 994 lesions. The ability to detect existing cancers (sensitivity), estimated from all studies, resulted to be about 98 % while the ability to recognise the normal condition in the absence of any false-positive finding (specificity), estimated from six studies reporting raw data, was about 58 %. The majority of included studies were judged to have examined very selected populations. Mean cancer size, reported only in three studies, was 21.2 mm. The authors concluded that high-quality studies are required to assess the CESM accuracy. In practice, CESM still deserves evaluation and the results of this meta-analysis cannot be considered as conclusive. Interestingly, two recent studies confirmed a high sensitivity of CESM (94–95 %) with higher values of specificity: 81 % in the symptomatic setting [35] and 74 % in the post-screening assessment [36].

On the basis of still preliminary results, CESM can be considered as *an alternative to contrast-enhanced MRI* in the case of contraindications to MRI (including the presence of MRI-unsafe devices in the patient’s body, claustrophobia and obesity preventing the patient from entering the magnet) or to gadolinium-based contrast injection as well as local conditions of difficult MRI availability [9, 10] due to interesting results obtaining by comparing CESM and MRI in the same patients [37, 38].

Note J. It is important to note that iodinated contrast agents are frequently used in clinical practice, mostly intravenously injected for contrast-enhanced computed tomography. There are contraindications (history of allergic reactions, renal failure) and possible side effects that require discussion with the patient and the signature of an informed written consent. Thus, the injection of iodinated contrast agents for mammography requires the same precautions used for other contrast-enhanced x-ray-based examination [39, 40]. Before the examination, the radiologist will clarify the risks and benefits associated with the intravenous injection of iodinated contrast agents.

## Frequently asked questions (FAQs)

### How painful is breast compression for mammography?

Mammography is tolerated well by the vast majority of women. In particular, it is painless for about 40–50 % of women, a little painful for 40 %, rather painful for 12 % and very painful only for 4 %. Pain disappears immediately after the procedure for 76 % of the women, while it lasts several minutes for 13 %, several hours for 7 % and more than 1 day for 4 % [11]. However, the advantages of compression are clear, and unnecessary pain may sometimes be avoided by suitable scheduling (see Note C). The radiographer will guide you through all the steps of the examination and will take care of minimising the discomfort during breast compression.

### When should the first mammogram be done? What are the time intervals for further examinations?

Different recommendations are issued by different radiological and cancer societies as well by health authorities and governmental bodies. There is a general agreement on the usefulness of screening mammography from 50 to 70 years of age, with a time interval depending on several factors described above. Extension from about 40–45 to about 75 is now adopted by several screening programmes. When starting at 40, a 1-year interval can be recommended up to 45–50, considering the probable higher density and the possible faster growth of the tumour. After 50, the optimal interval may be decided based on personal history and breast density. If you have symptoms, mammography may be necessary for you at any age. If you are a woman with an increased risk for breast cancer (gene mutation carrier, multiple breast/ovarian cancer in the family), screening should start before age 40, according to your personal calculated risk level, access to special screening programmes, and other factors.

Note K. If you are invited to attend an organised screening programme, follow the programme’s planned interval. If you have any doubts about this time interval, or the usefulness of ultrasound as a supplemental screening method, consult your radiologist. If there are a high number of incidences of breast cancer in your family, especially at a young age and before menopause, you may need to have a screening with MRI [9, 10]: consult your radiologist and/or a specialised centre (e.g. a family cancer clinic). Information on indications to MRI are available in a EUSOBI dedicated paper [10].

### What about screening mammography for women over 75?

The continuous increase in life expectancy prevents defining a clear cut upper age limit for screening mammography. A general suggestion is to continue screening with mammography for elderly women as long as their health is not significantly compromised by illness that drastically reduces life expectancy [41, 42]. Discuss this decision with your radiologist.

### Can women with breast implants or breast reconstruction undergo mammography?

Yes, in the majority of cases they can. Special views with back placement of the implant are commonly needed, as well as specific technical expertise by the radiographer. Exceptions where mammography cannot be performed are breast reconstructions after complete gland tissue removal. Mammography limitations due to the presence of implants can be counteracted by an accurate clinical breast examination and breast ultrasound.

Note L. Always tell the radiologist and/or the radiographer if you have breast implants.

### Is x-ray radiation from mammography dangerous?

The x-ray radiation associated with a mammogram is low. See in this article the section “Radiation exposure from mammography” for a comparison between the risk of radiation-induced breast cancer and the reduction of breast cancer mortality due to mammography.

### What is the role of new technologies like tomosynthesis and CESM?

The role of these new technologies is to help in the detection and diagnosis of breast cancers. Tomosynthesis is commonly accepted as an effective tool for evaluation of symptomatic patients and suspicious findings at screening mammography. Large studies in the screening setting showed that tomosynthesis allows the identification of more cancers than mammography and potentially reduces the number of women recalled for benign findings. So far, CESM has been evaluated in a limited number of small studies. It provides useful information of suspicious lesions, increasing the visibility of malignant lesions, in particular in women with dense breasts, and can be an alternative to contrast-enhanced MRI, especially in the case of contraindications to MRI or to gadolinium-based contrast injection as well as of difficult MRI availability.

## References

[1] Sardanelli F, Helbich TH, European Society of Breast Imaging (2012). Mammography: EUSOBI recommendations for women’s information. Insights Imaging.

[2] Sardanelli F, Aase H, Álvarez M et al. (2016) Position paper on screening for breast cancer by the European Society of Breast Imaging and 30 national breast radiology bodies from Austria, Belgium, Bosnia and Herzegovina, Bulgaria, Croatia, Czech Republic, Denmark, Estonia, Finland, France, Germany, Greece, Hungary, Iceland, Ireland, Israel, Italy, Lithuania, Moldova, The Netherlands, Norway, Poland, Portugal, Romania, Serbia, Slovakia, Spain, Sweden, Switzerland, and Turkey. Eur Radiol Nov 2 [Epub ahead of print] 10.1007/s00330-016-4612-z PMC5486792 27807699

[3] Feig SA (2014). Screening mammography benefit controversies: sorting the evidence. Radiol Clin N Am.

[4] Lauby-Secretan B, Scoccianti C, Loomis D, International Agency for Research on Cancer Handbook Working Group (2015). Breast Cancer Screening—viewpoint of the IARC Working Group. N Engl J Med.

[5] Saadatmand S, Bretveld R, Siesling S, Tilanus-Linthorst MMA (2015). Influence of tumour stage at breast cancer detection on survival in modern times: population based study in 173,797 patients. BMJ.

[6] Kaplan HG, Malmgren JA, Atwood MK, Calip GS (2015). Effect of treatment and mammography detection on breast cancer survival over time: 1990–2007. Cancer.

[7] Prummel MV, Muradali D, Shumak R (2016). Digital compared with screen-film mammography: measures of diagnostic accuracy among women screened in the Ontario breast screening program. Radiology.

[8] Perry N, Broeders M, de Wolf C et al. (2006) European guidelines for quality assurance in breast cancer screening and diagnosis. Fourth Edition. Available at: http://www.euref.org/european-guidelines. Accessed on Sept 10, 2016 10.1093/annonc/mdm481 18024988

[9] Sardanelli F, Boetes C, Borisch B (2010). Magnetic resonance imaging of the breast: recommendations from the EUSOMA working group. Eur J Cancer.

[10] Mann RM, Balleyguier C, Baltzer PA, European Society of Breast Imaging (EUSOBI), with language review by Europa Donna–The European Breast Cancer Coalition (2015). Breast MRI: EUSOBI recommendations for women’s information. Eur Radiol.

[11] Drossaert CHC, Boer H, Seydel ER (2002). Monitoring women’s experiences during three rounds of breast cancer screening: results from a longitudinal study. J Med Screen.

[12] American College of Radiology (ACR) Breast Imaging Reporting and Data System Atlas (BI-RADS Atlas). Reston, Va, USA: American College of Radiology; 2013. At: http://www.acr.org/Quality-Safety/Resources/BIRADS. Accessed on 9 Sept 2016.

[13] Törnberg S, Kemetli L, Ascunce N (2010). A pooled analysis of interval cancer rates in six European countries. Eur J Cancer Prev.

[14] Carbonaro LA, Azzarone A, Paskeh BB (2014). Interval breast cancers: absolute and proportional incidence and blinded review in a community mammographic screening program. Eur J Radiol.

[15] Yaffe MJ, Mainprize JG (2011). Risk of radiation-induced breast cancer from mammographic screening. Radiology.

[16] Hauge IH, Pedersen K, Olerud HM, Hole EO, Hofvind S (2014). The risk of radiation-induced breast cancers due to biennial mammographic screening in women aged 50–69 years is minimal. Acta Radiol.

[17] Biesheuvel C, Barratt A, Howard K (2007). Effects of study methods and biases on estimates of invasive breast cancer overdetection with mammography screening: a systematic review. Lancet Oncol.

[18] Paci E, Broeders M, Hofvind S, Puliti D, Duffy SW, EUROSCREEN Working Group (2014). European breast cancer service screening outcomes: a first balance sheet of the benefits and harms. Cancer Epidemiol Biomarkers Prev.

[19] Semturs F, Sturm E, Gruber R, Helbich TH (2010). Physical aspects of different tomosynthesis systems. Radiologe.

[20] Diekmann F, Bick U (2011). Breast tomosynthesis. Semin Ultrasound CT MR.

[21] Kopans DB (2014). Digital breast tomosynthesis from concept to clinical care. AJR Am J Roentgenol.

[22] Skaane P, Bandos AI, Eben EB (2014). Two-view digital breast tomosynthesis screening with synthetically reconstructed projection images: comparison with digital breast tomosynthesis with full-field digital mammographic images. Radiology.

[23] Svahn TM, Houssami N, Sechopoulos I, Mattsson S (2015). Review of radiation dose estimates in digital breast tomosynthesis relative to those in two-view full field digital mammography. Breast.

[24] Skaane P, Bandos AI, Gullien R et al (2013) Comparison of digital mammography alone and digital mammography plus tomosynthesis in a population-based screening program. Radiology 267:47–56 10.1148/radiol.12121373 23297332

[25] Skaane P, Bandos AI, Gullien R (2013). Prospective trial comparing full-field digital mammography (FFDM) versus combined FFDM and tomosynthesis in a population-based screening programme using independent double reading with arbitration. Eur Radiol.

[26] Ciatto S, Houssami N, Bernardi D (2013). Integration of 3D digital mammography with tomosynthesis for population breast-cancer screening (STORM): a prospective comparison study. Lancet Oncol.

[27] Lång K, Andersson I, Rosso A, Tingberg A, Timberg P, Zackrisson S (2016). Performance of one-view breast tomosynthesis as a stand-alone breast cancer screening modality: results from the Malmö Breast Tomosynthesis Screening Trial, a population-based study. Eur Radiol.

[28] Houssami N (2015). Digital breast tomosynthesis (3D-mammography) screening: data and implications for population screening. Expert Rev Med Devices.

[29] McDonald ES, Oustimov A, Weinstein SP, Synnestvedt MB, Schnall M, Conant EF (2016). Effectiveness of digital breast tomosynthesis compared with digital mammography: outcomes analysis from 3 years of breast cancer screening. JAMA Oncol.

[30] Fallenberg EM, Dromain C, Diekmann F (2014). Contrast-enhanced spectral mammography: does mammography provide additional clinical benefits or can some radiation exposure be avoided?. Breast Cancer Res Treat.

[31] Lobbes MBI, Lalji U, Houwers J (2014). Contrast-enhanced spectral mammography in patients referred from the breast cancer screening programme. Eur Radiol.

[32] Knogler T, Homolka P, Hörnig M (2016). Contrast-enhanced dual energy mammography with a novel anode/filter combination and artifact reduction: a feasibility study. Eur Radiol.

[33] Jeukens CRLPN, Lalji UC, Meijer E et al (2014) Radiation exposure of contrast-enhanced spectral mammography compared with full-field digital mammography. Invest Radiol 49:659–665 10.1097/RLI.0000000000000068 24872005

[34] Tagliafico AS, Bignotti B, Rossi F (2016). Diagnostic performance of contrast-enhanced spectral mammography: systematic review and meta-analysis. Breast.

[35] Tennant SL, James JJ, Cornford EJ et al (2016) Contrast-enhanced spectral mammography improves diagnostic accuracy in the symptomatic setting. Clin Radiol 71:1148–1155 10.1016/j.crad.2016.05.009 27296475

[36] Tardivel AM, Balleyguier C, Dunant A (2016). Added value of contrast-enhanced spectral mammography in postscreening assessment. Breast J.

[37] Jochelson MS, Dershaw DD, Sung JS (2013). Bilateral contrast-enhanced dual-energy digital mammography: feasibility and comparison with conventional digital mammography and MR imaging in women with known breast carcinoma. Radiology.

[38] Fallenberg EM, Dromain C, Diekmann F (2014). Contrast-enhanced spectral mammography versus MRI: Initial results in the detection of breast cancer and assessment of tumour size. Eur Radiol.

[39] Stacul F, van der Molen AJ, Reimer P, Contrast Media Safety Committee of European Society of Urogenital Radiology (2011). Contrast induced nephropathy: updated ESUR Contrast Media Safety Committee guidelines. Eur Radiol.

[40] Morcos SK, Bellin MF, Thomsen HS, Contrast Media Safety Committee of European Society of Urogenital Radiology (2008). Reducing the risk of iodine-based and MRI contrast media administration: recommendation for a questionnaire at the time of booking. Eur J Radiol.

[41] Simon MS, Wassertheil-Smoller S, Thomson CA (2014). Mammography interval and breast cancer mortality in women over the age of 75. Breast Cancer Res Treat.

[42] Oeffinger KC, Fontham ET, Etzioni R et al (2015) Breast cancer screening for women at average risk: 2015 guideline update from the American Cancer Society. JAMA 314:1599–1614 10.1001/jama.2015.12783 PMC4831582 26501536

